# Health-Related Quality of Life Among Older Adults in Kazakhstan During the Pandemic

**DOI:** 10.1093/geroni/igaf122.3252

**Published:** 2025-12-31

**Authors:** Assel Izekenova, Aigulsum Izekenova, Yineng Chen, Dinara Sukenova, Fei Sun

**Affiliations:** Michigan State University, East Lansing, Michigan, United States; Asfendiyarov Kazakh National Medical University, Almaty, Almaty, Kazakhstan; University at Albany, Albany, New York, United States; Asfendiyarov Kazakh National Medical University, Almaty, Almaty, Kazakhstan; Michigan State University, East Lansing, Michigan, United States

## Abstract

The COVID-19 pandemic has significantly impacted the well-being of older adults, raising concerns about their quality of life (QOL). This study examines the factors influencing QOL among older adults in Kazakhstan during the pandemic, specifically focusing on health-related outcomes. A cross-sectional survey was conducted from June to July 2022 with 445 community-dwelling individuals aged 60 and above. The Older People’s Quality of Life (OPQOL) scale was used to assess QOL, while psychological distress and loneliness were measured using the PHQ-4 and UCLA-3 scales, respectively. Univariate and multivariate regression analyses were applied to explore associations between QOL and demographic characteristics, pandemic-related experiences, psychological distress, and loneliness. Results revealed a mean OPQOL total score of 124.1 (SD = 11.3) and a mean health-related sub-score of 13.8 (SD = 2.9). Higher QOL was significantly associated with the absence of chronic diseases (p < 0.001), Kazakh ethnicity (p = 0.003), and minimal psychological distress (p < 0.001). Participants who downplayed concerns about COVID-19 tended to report better overall and health-related QOL (p ≤ 0.005). Additionally, younger age (p ≤ 0.031), better self-reported health (p < 0.001), and lower concern for family members’ infection risk (p = 0.024) were linked to higher health-related QOL scores. These findings emphasize the role of health status, psychological resilience, and perceptions of the pandemic in shaping older adults’ QOL. Targeted interventions addressing health disparities and pandemic-related stress may improve well-being among older populations in Kazakhstan.

